# Genome-wide DNA methylation profile of developing deciduous tooth germ in miniature pigs

**DOI:** 10.1186/s12864-016-2485-9

**Published:** 2016-02-24

**Authors:** Yingying Su, Zhipeng Fan, Xiaoshan Wu, Yang Li, Fu Wang, Chunmei Zhang, Jinsong Wang, Jie Du, Songlin Wang

**Affiliations:** Molecular Laboratory for Gene Therapy and Tooth Regeneration, Beijing Key Laboratory of Tooth Regeneration and Function Reconstruction, Capital Medical University School of Stomatology, Tian Tan Xi Li No.4, Beijing, 100050 China; Laboratory of Molecular Signaling and Stem Cells Therapy, Beijing Key Laboratory of Tooth Regeneration and Function Reconstruction, Capital Medical University School of Stomatology, Tian Tan Xi Li No.4, Beijing, 100050 China; Department of Biochemistry and Molecular Biology, Capital Medical University School of Basic Medical Sciences, You An Men Wai Xi Tou Tiao No.10, Beijing, 100069 China; Department of Physiology and Pathophysiology, Beijing An Zhen Hospital the Key Laboratory of Remodeling-Related Cardiovascular Diseases, School of Basic Medical Sciences, Capital Medical University, You An Men Wai Xi Tou Tiao No.10, Beijing, 100069 China

**Keywords:** DNA methylation, MeDIP-seq, Odontogenesis, Biomineralization, Miniature pig

## Abstract

**Background:**

DNA methylation is an important epigenetic modification critical to the regulation of gene expression during development. To date, little is known about the role of DNA methylation in tooth development in large animal models. Thus, we carried out a comparative genomic analysis of genome-wide DNA methylation profiles in E50 and E60 tooth germ from miniature pigs using methylated DNA immunoprecipitation-sequencing (MeDIP-seq).

**Results:**

We observed different DNA methylation patterns during the different developmental stages of pig tooth germ. A total of 2469 differentially methylated genes were identified. Functional analysis identified several signaling pathways and 104 genes that may be potential key regulators of pig tooth development from E50 to E60.

**Conclusions:**

The present study provided a comprehensive analysis of the global DNA methylation pattern of tooth germ in miniature pigs and identified candidate genes that potentially regulate tooth development from E50 to E60.

**Electronic supplementary material:**

The online version of this article (doi:10.1186/s12864-016-2485-9) contains supplementary material, which is available to authorized users.

## Background

Tooth development is characterized by a sequential and reciprocal series of inductive signals transmitted between the epithelium and mesenchyme [1, 2]. A number of transcription factors, signaling molecules, growth factor receptors, and extracellular matrix molecules have been identified as participating in the regulation of odontogenesis patterning and differentiation processes [1]. Currently, almost all of our understanding of molecular mechanisms controlling tooth formation and mineralization has come from studies in mice [3–5], whose teeth are significantly different from those of humans in regards to both morphology and number, with only one set of dentition and an absence of canines and premolars [6]. Recently, our group used miniature pigs, which resemble humans in anatomy, physiology, pathophysiology, and development, as an animal model to study the complicated mechanism of tooth development. We identified the characteristic patterns of the spatiotemporal morphogenesis of successional teeth in miniature pigs [7] and mapped the mRNA and microRNA expression profiles [8, 9]. Our results identified differential gene expression patterns in different developmental stages and a spatio-temporal pattern of down-regulation during tooth development [8], suggesting that tooth formation is the result of tight control by a sequence of molecular networks that act at particular places and times. Despite numerous studies leading to the discovery of genetic mechanisms underlying the expression of specific genes during different stages of tooth development [3], recent studies suggest that epigenetic mechanisms also participate in tooth development [10–12].

DNA methylation is one of the best-studied epigenetic modifications and regulates a variety of processes, including embryonic development, cellular differentiation, tissue-specific gene expression, genomic imprinting, X chromosome in activation, and chromosome stability [13–15]. In vertebrates, DNA methylation occurs predominantly at cytosine residues within CpG dinucleotides [16]. CpG-rich regions are referred to as CpG islands (CGIs), and are located in nearly 40 % of the promoters of mammalian genes [17]. CGIs usually have global unmethylated patterns [18]. Unmethylated CGIs in the promoter regions are normally associated with gene expression, whereas methylated CGIs usually result in gene silencing [13, 19]. Although DNA methylation has been widely accepted as a key mechanism of transcriptional regulation and a critical factor in the development of various organs [20–23], little is known about the normal developmental changes in DNA methylation during odontogenesis.

In the present study, we characterized the genome-wide temporal dynamics of DNA methylation in developing deciduous molars in miniature pigs using methylated DNA immunoprecipitation combined with high-throughput sequencing (MeDIP-seq) to further investigate the role of DNA methylation in gene expression during tooth development. We revealed the landscape of the methylome and identified the potential role of DNA methylation in gene expression in developing tooth germ. The results support utilizing the pig as a model organism for tooth development research.

## Results

### Global mapping of DNA methylation in the tooth germ of miniature pigs

We mapped the global DNA methylation status of tooth germ collected from E50 and E60 miniature pig embryos. After the removal of low-quality reads from raw MeDIP-seq data, an average of 4.6 Gb of clean reads were obtained per sample. Total reads were mapped to the reference genome, and mapping rates ranged from 84.30 to 85.55 %, of which 59.95–62.85 % were uniquely mapped to specific regions in the pig genome (Additional file 1: Table S1). Only uniquely mapped reads were used in further analysis.

Uniquely mapped reads were detected in all chromosomes (GGA1-18 and chromosome X) (Additional file 2: Figure S1). The genome coverage of the CG, CHG, and CHH sites negatively correlated with read depth; most regions had low sequencing depth, and a small number of regions had highs equencing depth (Additional file 3: Figure S2). The distribution of reads indifferent CG density regions was also analyzed, demonstrating that low CG density regions covered more uniquely mapped reads than any other region (Additional file 4: Figure S3).

**Fig. 1:**
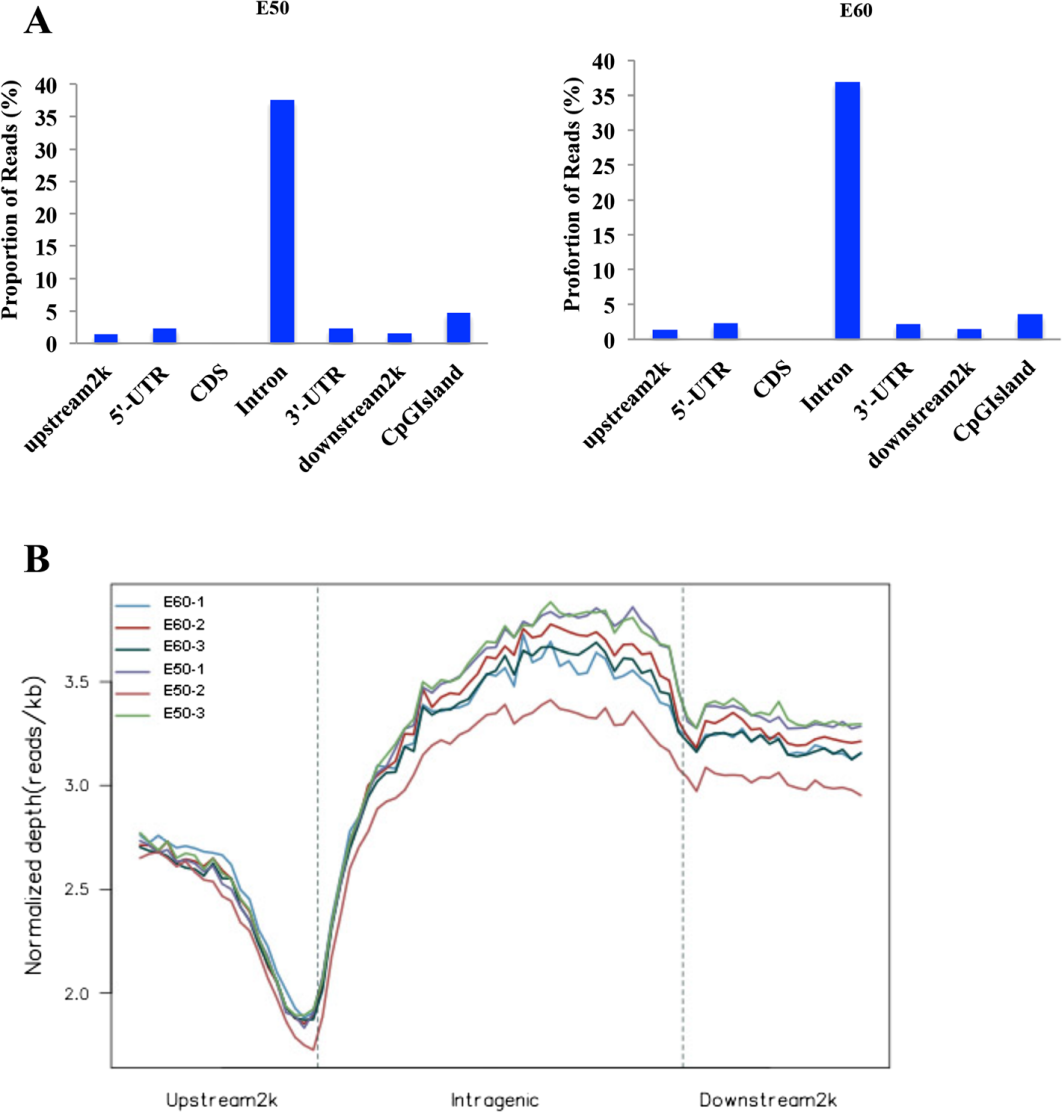
Distribution of unique mapped reads in different genomic regions. **a** The majority of reads were present in the intron regions. The x-axis shows the different gene regions. The y-axis is the percentage of unique mapped reads. **b** The gene was divided into three regions: a 2 kb region upstream of the transcription start site (TSS), the gene body from the TSS to the transcription termination site (TTS), and a 2 kb region downstream of the TTS. The DNA methylation level was lowest around the TSS

Different genomic regions exhibited different methylation patterns. The majority of reads were present in the intron regions, followed by CGIs (Fig. 1a). Within the gene body, a depletion of or decrease in reads occurred at the transcription start site (TSS) in both E50 and E60 tooth germ. In contrast, a gradual increase in reads occurred in the intragenic region (Fig.1b).

**Fig. 2:**
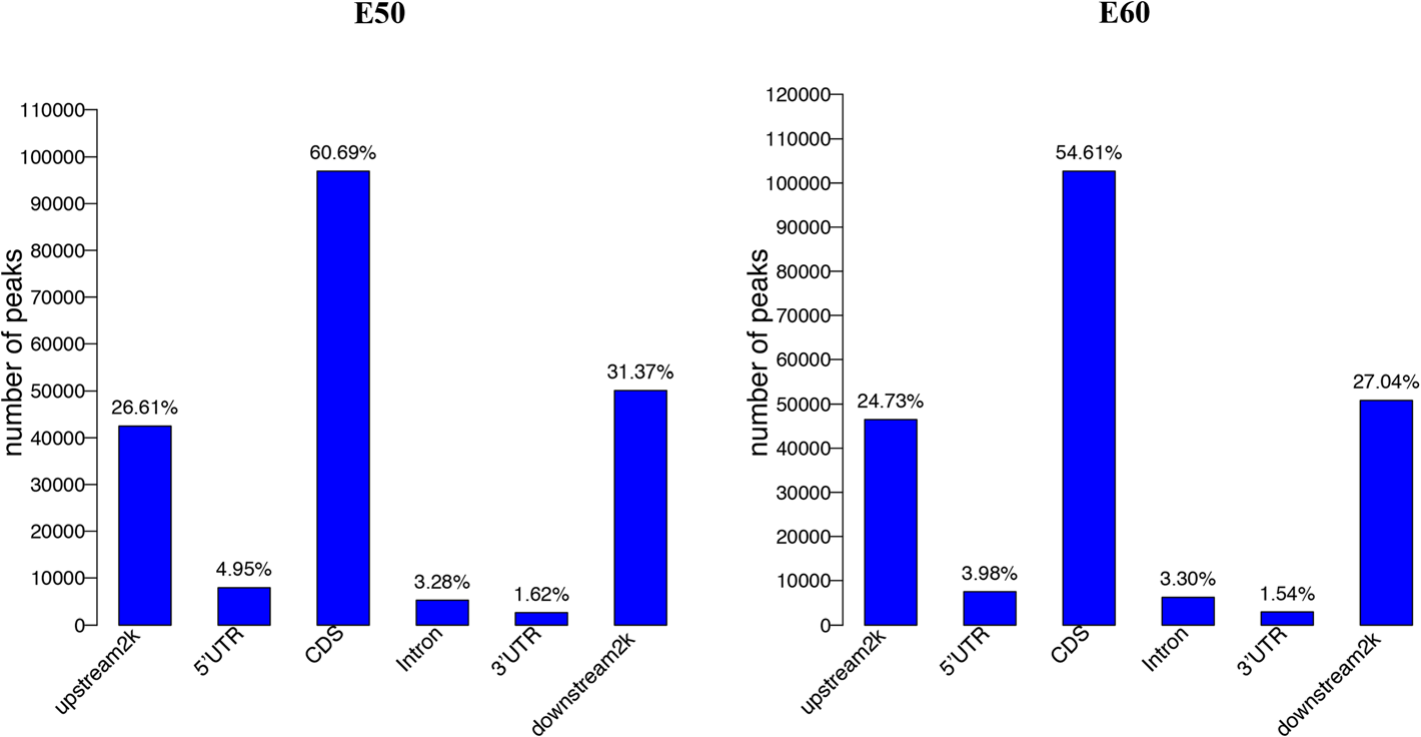
Distribution of DNA methylation peaks in different genomic regions. Peaks were defined as regions with sequencing tags more than 10 and *p* value < 10^−5^ with MACS. A major portion of peaks were present in the CDS region, followed by 2 kb downstream from the transcription termination site and2 kb upstream from the TSS, whereas the5’-UTR, intron, and 3’-UTRhad fewer peaks. The x-axis shows the different genomic regions. The y-axis is the number of peaks

Methylation peaks, referred to as methyl-cytosine-enriched regions, are important parameters for the identification of global DNA methylation status [24]. Using MACS1.4.0., we obtained 135,043–179,112 peaks, covering 6.97–7.94 % of the reference genome (Additional file 1: Table S2). We further analyzed the distribution of peaks in different components of the genome. A major portion of the peaks were present in the CDS region, followed by 2 kb downstream of the transcription termination site and 2 kb upstream of the TSS, whereas the 5’-UTR, intron, and 3’-UTRhad fewer peaks (Fig. 2). Here, 2 kb upstream of TSS was considered the proximal promoter.

### MeDIP-seq data validation by bisulfite sequencing

**Fig. 3:**
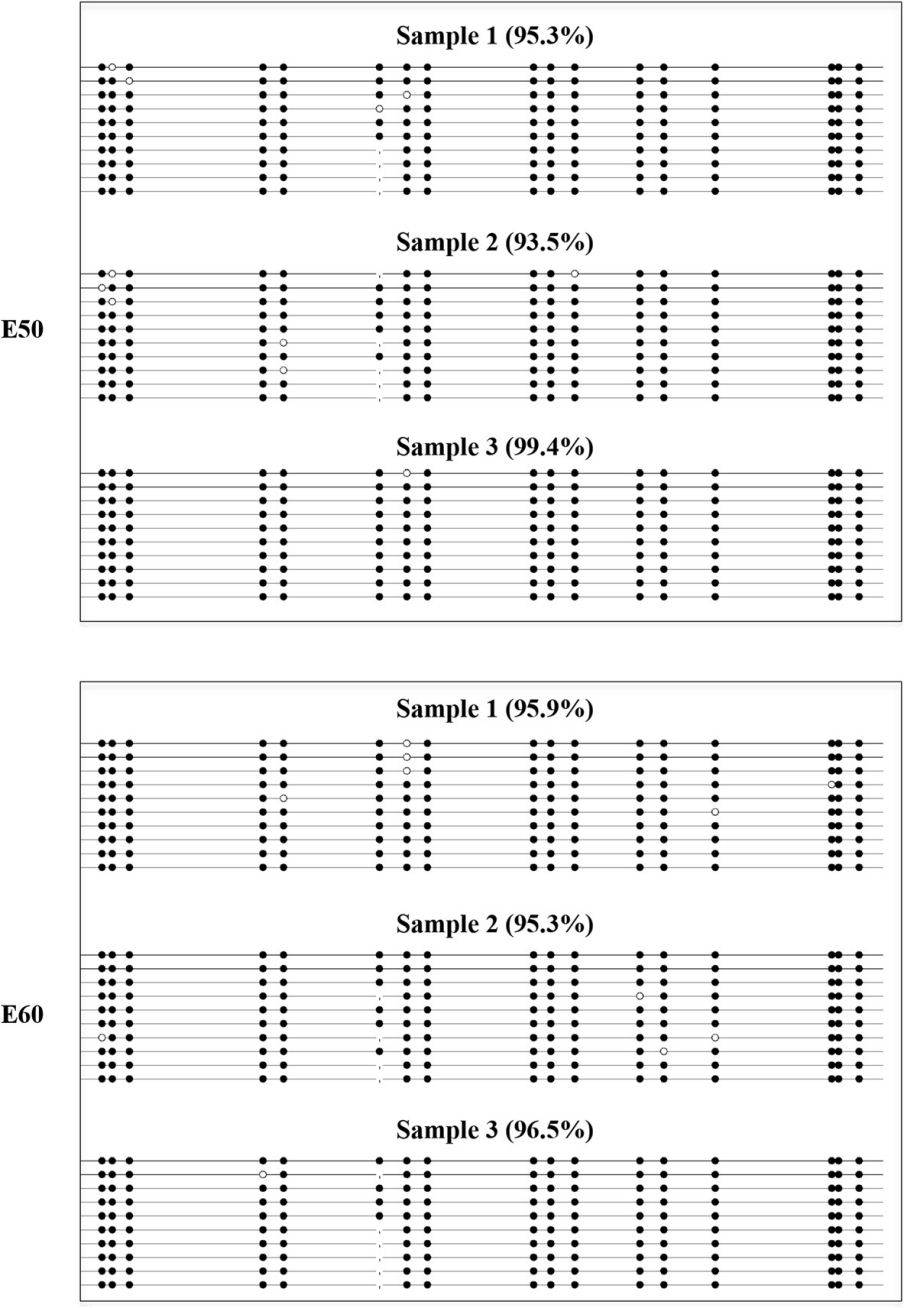
Validation of MeDIP-seq data by bisulfite sequencing (BSP). A highly methylated region obtained from MeDIP-seq data was randomly selected to verify its methylation pattern by BSP. Each line represents a single strand of DNA. Each circle represents a single CpG dinucleotide. Methylated CpGs are indicated by filled circles, whereas unmethylated CpGs are indicated by open circles

To validate the MeDIP-seq data, three regions with relatively high methylation were selected at random for bisulfite sequencing. The results obtained for the three gene regions were in accordance with the MeDIP-seq results. Moreover, great consistency was found among individuals in the same group (Fig. 3, Additional file 5: Figure S4).

### Differential DNA methylation in E50 and E60 tooth germ

**Fig. 4:**
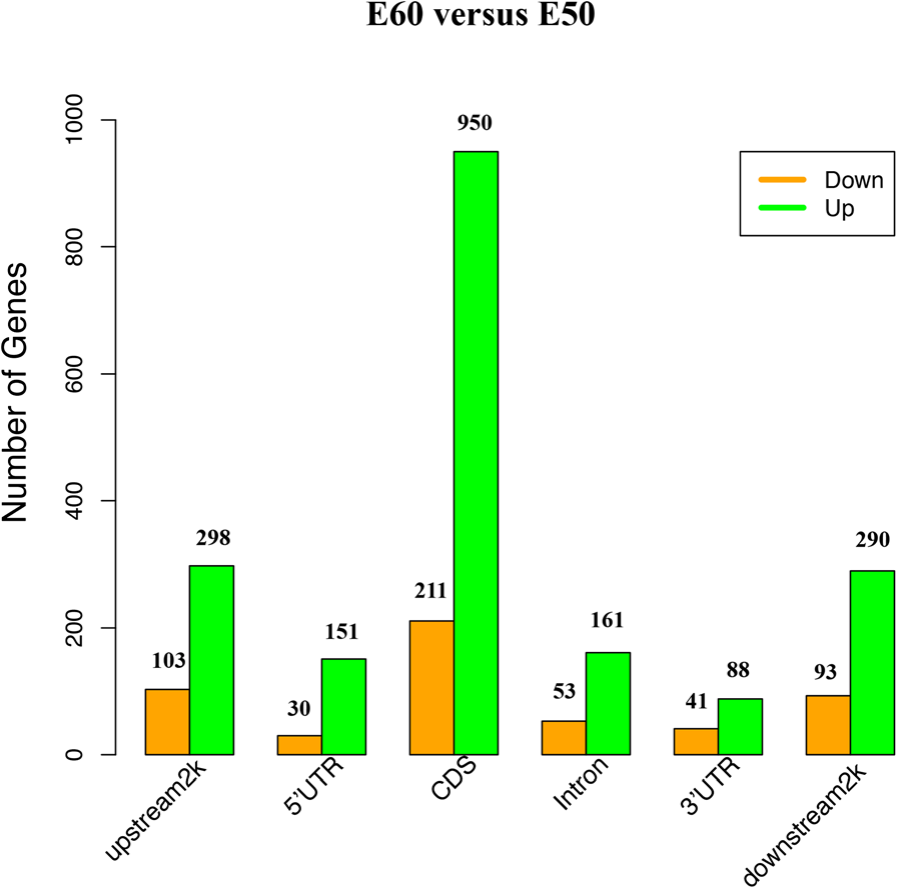
Distribution of differentially methylated genes in different genomic regions. A comparison of differentially methylated genes on E60 versus E50 shows more up-regulated genes than down-regulated genes. The number of differentially methylated genes is given at the top of each column

A comparison of differentially methylated regions (DMRs) between E50 and E60 tooth germ revealed 10,488 DMRs (Additional file 1: Table S3). Genes with methylation peaks in both the promoter and gene body regions were considered to be methylated genes [24]. Next, we identified genes containing DMRs in the two groups. A total of 2469 differentially methylated genes were identified: 401 differentially methylated in the 2 kb upstream (103 down-methylated genes, 298 up-methylated genes), 181 in the 5’UTR (30 down-methylated genes, 151 up-methylated genes), 1161 in the CDS (211 down-methylated genes, 950 up-methylated genes), 213 in the intron (53 down-methylated genes, 161 up-methylated genes), 129 in the3’UTR (41 down-methylated genes, 88 up-methylated genes), and 383 2 kb downstream (93 down-methylated genes, 290 up-methylated genes). More genes were up-methylated (*n* = 1938) than down-methylated (*n* = 531) in E60 tooth germ compared to E50 tooth germ (Fig. 4, Additional file 6).

### Analysis of functional categories of differentially methylated genes

**Fig. 5:**
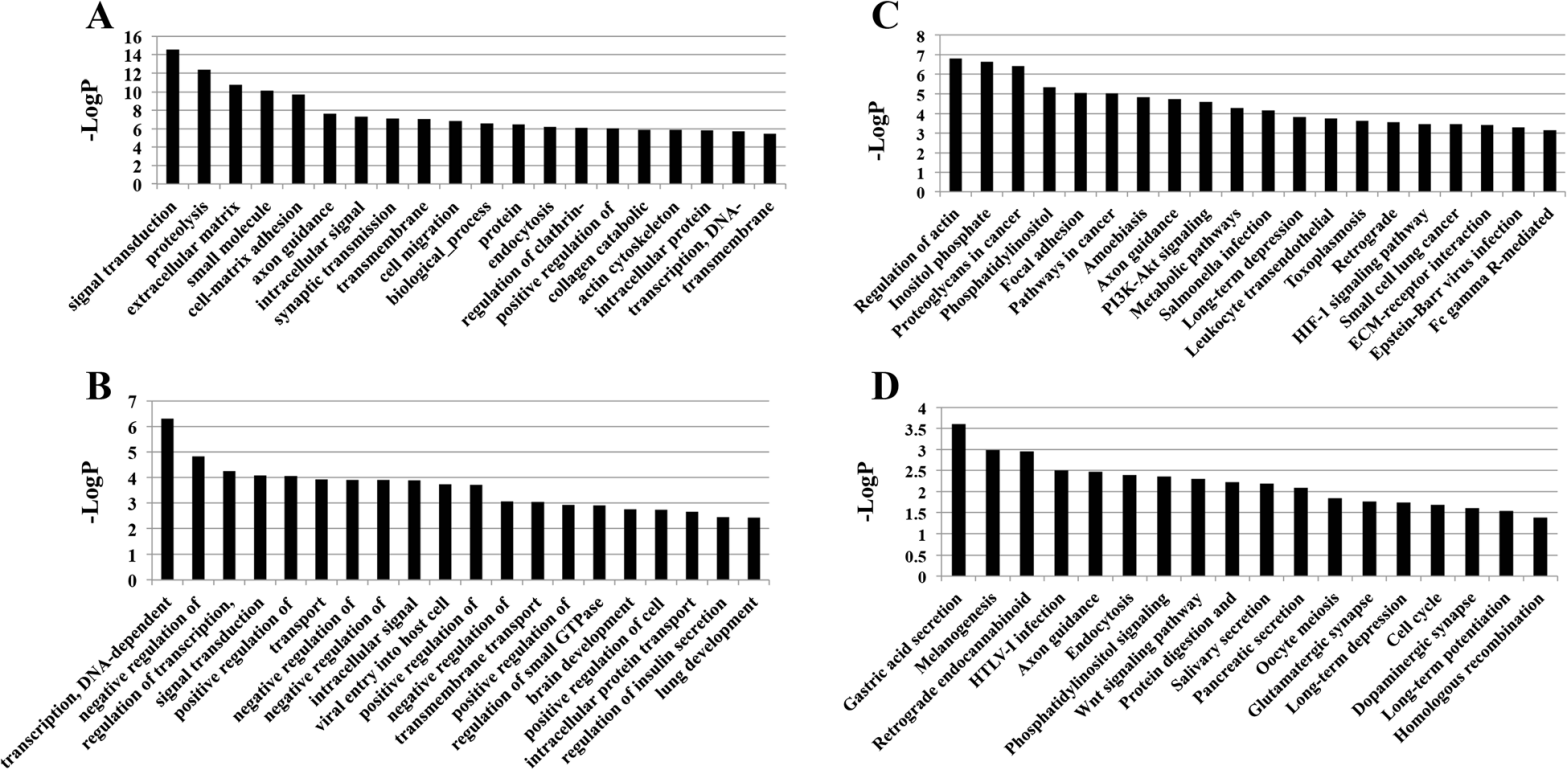
Gene ontology and pathway analysis of differentially methylated genes. **a** Top 20 significant GO groups involving up-regulated differentially methylated genes (*p* < 3E-06). **b** Top 20 significant GO groups involving down-regulated differentially methylated genes (*p* < 0.001). **c** Top 20 significant pathways involving up-regulated differentially methylated genes (*p* < 0.0005). **d** Top 20 significant pathways involving down-regulated differentially methylated genes (*p* < 0.05)

To identify the biological functions associated with differentially methylated genes at E60 versus E50, we performed Gene Ontology (GO) analysis using the DAVID program. GO assignments revealed that the up-methylated genes were mainly involved in 301 categories (*p* < 0.05, Additional file 7). The top 20 GO categories was showed in Fig. 5a including signal transduction, proteolysis, extracellular matrix organization, small molecule metabolic processes, cell-matrix adhesion, axon guidance, and intracellular signal transduction (*p* < 3E-06). The down-methylated genes strongly correlated with 180 categories (*p* < 0.05, Additional file 8). The top 20 included transcription (DNA-dependent), negative regulation of apoptosis, regulation of transcription (DNA-dependent), signal transduction, positive regulation of transcription (DNA-dependent), transport, and negative regulation of chromatin silencing at rDNA (*p* < 0.001, Fig. 5b).

### Pathway and path-net analysis of differentially methylated genes

To determine the significant pathways involved in differential methylation, we used the Kyoto Encyclopedia of Genes and Genomes (KEGG) pathway database to predict putative functions. The up-methylated genes were significantly enriched in 79 predicted pathways (*p* < 0.05, Additional file 9), the most significant of which was regulation of the actin cytoskeleton, inositol phosphate metabolism, proteoglycans in cancer, phosphatidylinositol signaling, and focal adhesion (*p* < 0.0005, Fig. 5c). The down-methylated genes were tightly related to 18 pathways, including gastric acid secretion, endocytosis, the wnt signaling pathway, and cell cycle (*p* < 0.05, Fig. 5d, Additional file 10).

**Fig. 6:**
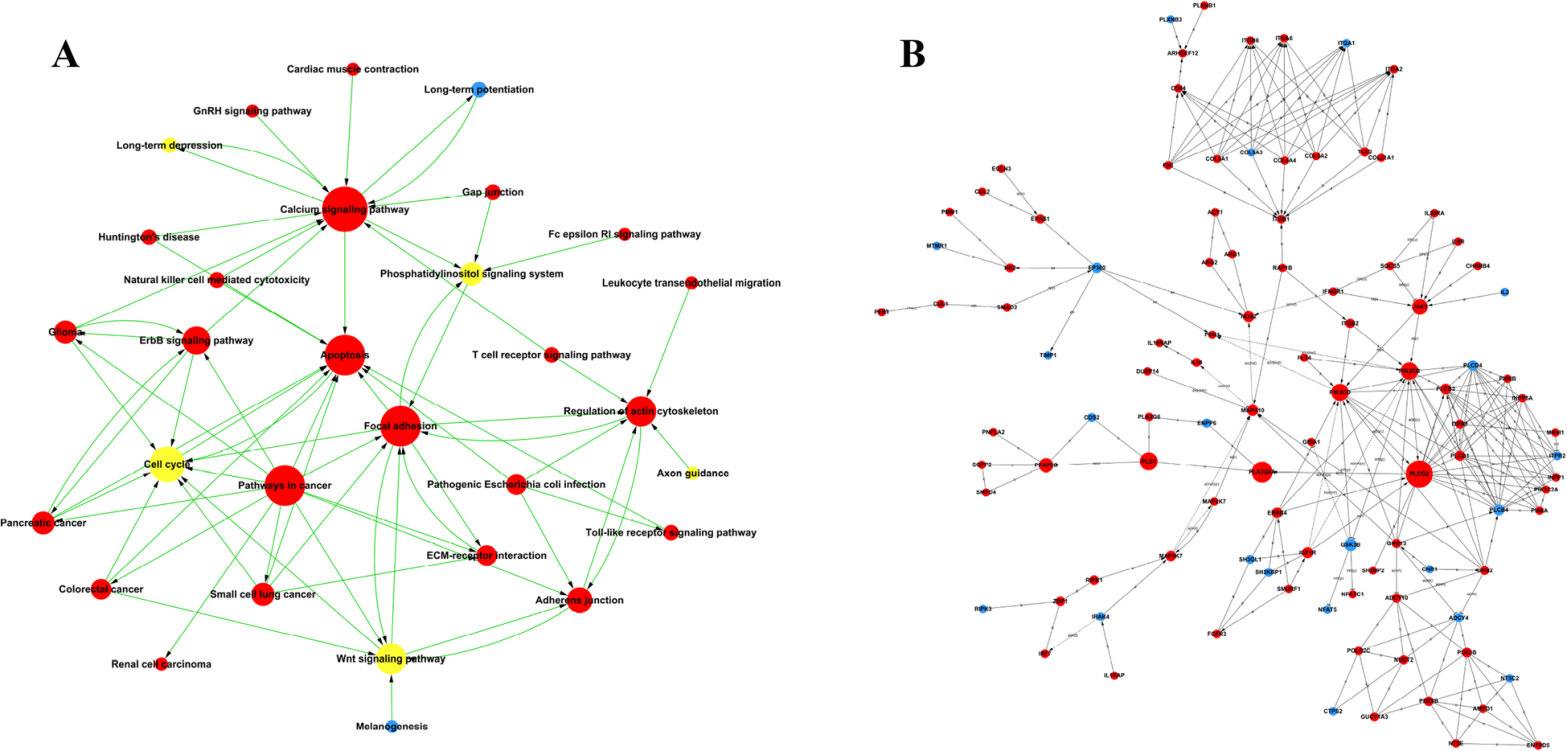
Path-net and gene-gene interaction analysis of differentially methylated genes. **a** Path-net analysis of differentially methylated genes. Cycle nodes represent pathways, the size of the node represents the power of the interrelation among the pathways, and the lines represent the interaction between the pathways. The more edges in a pathway, the more pathways connecting to it and the more central role it plays within the network. **b** Gene-gene interaction analysis of differentially methylated genes. Red circle nodes represent up-regulated genes, and blue circle nodes represent down-regulated genes. The size of the node represents degree, and lines represent interactions between the genes. A straight line represents combinations anda dotted line represents indirect effects. a, activation; b, binding; c, compound; inh, inhibition; u, ubiquination; s, state change; dep, dephosphorylation

To further understand the interactions among the significant pathways involving the up- and down-regulation of differentially methylated genes, an interaction net was built using path-net analysis. As shown in Fig. 6a, some differentially methylated genes were identified as being involved in key pathways during tooth morphogenesis, including the calcium signaling pathway, apoptosis, focal adhesion, pathways in cancer, regulation of the actin cytoskeleton, and ErbB signaling.

### Signal-net analysis

Gene signal transduction networks (Signal-net) were built to explore inter-gene signaling between the differentially methylated genes. A total of 104 genes were screened as potential core regulators of tooth development from E50 to E60 (Additional file 11). As shown in Fig. 6b, *PLCG2, PLA2G4A, PLD1, PIK3CB, PIK3CD, JAK2, PPAP2C, MAPK10* had higher betweenness centrality, degree, indegree, and outdegree, indicating that they may play crucial roles in odontogenesis. *GSK3*β, *ERBB4*, and *IGF1R*, which are involved in the wnt signaling pathway, ErbB signaling, and insulin-like growth factor (IGF) signaling, respectively, were also identified as core regulators that interact with other molecules in the signaling network.

## Discussion

This study provides a comprehensive analysis of genome-wide DNA methylation profiles in the developing tooth germ of miniature pigs. The results revealed analogous DNA methylation patterns to those of other species, from mammals to plants [24–31]. In the tooth germ of miniature pigs, the CGIs and promoters (around TSSs) remain hypomethylated, whereas the methylation levels in gene body regions were relatively high. In mammals, most CpG sites are methylated, and highly methylated sequences are usually found in the gene body, repeats, satellites, and non-repetitive intergenic regions [32]. However, CGIs in approximately 60 % of the promoters of human genes and TSSs are normally hypomethylated [33]. In general, the DNA methylation levels of CGIs and TSSs are associated with gene expression [34, 35]. Unmethylated CGIs in the promoters are essential for tissue-specific expression of corresponding genes in early embryos and later somatic cells [36]. The methylation patterns revealed in this study were consistent with those reported in previous studies.

The comparison of DNA methylation profiles between E60 and E50 tooth germ showed the different DNA methylation patterns in the different developmental stages of pig tooth germ. Pathway analysis of differentially methylated genes indicated that many tooth morphogenesis-related pathways were involved. As expected, several significant pathways were obtained, including those involved in the actin cytoskeleton, focal adhesion, wnt signaling, and calcium signaling. Among these pathways, the wnt pathway is one of the most important during tooth morphogenesis. From E50 to E60, pig tooth germ progressed from the bell stage to secretory stage. At the bell stage, the enamel- and dentine-forming cells differentiate. Initially, the mesenchymal cells differentiate into odontoblasts that secrete the dentine matrix. Subsequently, the adjacent epithelial cells differentiate into ameloblasts and secrete enamel matrix. The odontoblasts and ameloblasts then control the phases of mineralization of enamel and dentine. At the secretory stage, tooth development is characterized by the transformation of soft gel-like extracellular matrices into mineralized structures of the skeleton [37]. The actin cytoskeleton, focal adhesion, and calcium signaling pathway play key roles in cytoskeletal organization [38]; the pathway analysis implied that they likely contribute to tooth biomineralization. The pathway profiles involving differentially methylated genes provided insight into understanding the maturation mechanisms of tooth germ in miniature pigs.

Signal-net analysis screened several key genes that may contribute to the transformation of soft tissue into hard tissue, including *PLCG2, PIK3CB, PIK3CD*, *GSK3*β*, MAPK10, ERBB4,* and *IGF1R*. The phospholipase C gamma-2 (*PLCG2*) gene is important for intracellular signal transduction pathways because it encodes an enzyme that plays a crucial role in the generation of second messengers following the hydrolysis of phosphatidylinositol 4, 5-bisphosphate [39]. GSK3β, a negative regulator of the wnt canonical pathway, plays a crucial role in tooth morphogenesis, and inhibition of GSK3β could delay the differentiation of ameloblasts and odontoblasts [40]. PIK3CB and PIK3CD belong to the phosphoinositide 3-kinase (PI3K) family. PI3K and MAPK family members are involved in the regulation of many cellular processes, such as proliferation, migration, survival, and apoptosis, as well as tooth development [41–43]. ERBB4 and IGFIR were also identified to be associated with odontogenesis and ameloblast differentiation [44–46]. The results indicate novel epigenetic mechanisms regulating tooth mineralization.

## Conclusions

In summary, this study provided a comprehensive analysis of DNA methylation profiles in developing tooth germ from miniature pigs during biomineralization and identified 104 differentially methylated genes that may be potential core regulators of tooth development from E50 to E60. The genes and pathways screened suggest strong candidates for in-depth studies of the epigenetic mechanisms underlying tooth development in miniature pigs.

## Methods

### Animals and tissue collection

The Wuzhishan miniature pigs used in this study were purchased from Kexing Laboratory Animal Company of Beijing, China. This study was conducted in accordance with the recommendations of the Regulations for the Administration of Affairs Concerning Experimental Animals (Ministry of Science and Technology, China). All the animal experiments were approved by the Animal Care and Use Committees of Capital Medicine University under the permit (Approval Number CMU-B20100106). The last deciduous mandibular molar germs at embryonic day 50 (E50) and E60 which corresponded to early and late bell stages were used in this study. Pregnant miniature pigs were anesthetized and sacrificed as previously described [8]. The tooth germs were isolated under a microscope, frozen immediately in liquid nitrogen, and stored separately at−80 °C until DNA extraction.

### MeDIP sequencing

For each developmental stage, the tooth germs were isolated separately from three randomly selected embryos with a specific sex (male) as biological replicates. MeDIP DNA libraries were constructed following the protocol as described previously [47]. In brief, genomic DNA was extracted using the DNeasy Blood & Tissue Kit (Qiagen, Hilden, Germany) according to the manufacturer’s instructions. Five microgram DNA was sonicated to fragments ranging from 100 to 500 bp. Subsequently, DNA underwent end-repair, the generation of 3’-dA overhangs, and adaptor ligation steps using Paired-End DNA Sample Prep kit (Illumina, San Diego, CA, USA). Adaptor-ligated DNA was then immunoprecipitated by anti-5-methylcytosine monoclonal antibody (Diagenode, NJ, USA). The enriched methylated fragments and 10 % input DNA were purified on DNA Clean & Concentrator-5 columns (Zymo, CA, USA) according to the manufacturer’s manuals. Enriched fragments were amplified by adaptor-mediated PCR, the products were quantified on Agilent 2100 Analyzer (Agilent Technologies, Santa Clara, CA, USA), and MeDIP library was sequenced on an Illumina HiSeq 2000 Sequencing System by Beijing Genomics Institute (BGI, Shenzhen, China). After the completion of sequencing run, raw data was processed by the Illumina base-calling pipeline.

### Bioinformatic analysis

Raw sequencing data were first processed to filter out low-quality reads that containing more than 5 ‘N’s or over 50 % of the sequence with low quality value (Phred score < 5). The MeDIP-seq data were aligned to the UCSC pig reference genome (Sscrofa9.2, http://hgdownload.cse.ucsc.edu/goldenPath/susScr2/chromosomes/.), allowing up to two mismatches using SOAP2 (Version 2.21) [48]. For each sample, we analyzed the distributions of MeDIP-Seq reads on the pig genome, in CGIs, gene region and different CG density regions, as well as the genome coverage of the CG, CHG, and CHH (with H being A, T or C) sites under different sequencing depths. The distribution of peaks in different genome components in each group, including the upstream 2 kb, 5’-untranslated region (UTR), 3’-UTR, CDS, intron, and downstream 2 kb was also analyzed.

The genomic differentially methylated regions (DMRs) between E50 and E60 groups were identified using the method described previously [49]. The number and the distribution of DMRs between two groups were analyzed. All DMR-containing genes were subsequently processed to functional enrichment analysis of GO and KEGG pathway using the DAVID (the Database for Annotation, Visualization and Integrated Discovery) web server. For GO and pathway analysis, Parent–child-Intersection method was used for enrichment analysis and Benjamini-Hochberg was used for multiple tests correction.

### Bisulfite sequencing PCR (BSP)

There were three individual samples in E50 and E60 groups, respectively. Three relatively high methylated gene regions were chosen to validate MeDIP-seq data with BSP using individual samples. The primers for the three gene regions were listed in Additional file 1: Table S4. Bisulfite modification of 500 ng of genomic DNA was performed using Methyl Code Bisulfite Conversion Kit (Invitrogen, USA). The bisulfite-treated DNA was amplified by PCR in 25 μl reaction mixtures. The cycle program was set to 3 min at 95 °C, one cycle; 30 s at 95 °C, 40 s at 50 °C, 40 s at 72 °C, 40 cycles; and a final extension 10 min at 72 °C. PCR products were gel-purified with the E.Z.N.A. Gel Extraction Kit (Omega) and cloned into the pMD18-T vector (Takara). Positive clones were randomly collected for DNA sequencing. The sequencing data and non-CpG-C–T conversion rates were analyzed using BiQAnalyzer software. Methylation status of the target sequence was displayed as the percentage of methylated CpGs of the total number of CpGs.

### Availability of supporting data

The data sets supporting the results of this article are included within the article and its additional files.

## Additional files

Additional file 1: Table S1. Date generated by MeDIP-seq. **Table S2** Numbers of differentially methylated regions in different gene components. **Table S3** The information of primers for bisulfite sequencing. (DOCX 21 kb) Additional file 2: Figure S1. Chromosome distribution of reads in the E50 and E60 tooth germ. (TIF 1845 kb) Additional file 3: Figure S2. Genome coverage of the CG, CHG, and CHH sites under different sequencing depth. (TIF 617 kb) Additional file 4: Figure S3. Distribution of MeDIP-Seq reads in different CG density regions. (TIF 504 kb) Additional file 5: Figure S4. The validation of MeDIP-seq data by bisulfite sequencing (BSP). (TIF 1800 kb) Additional file 6:
**The list of differentially methylated genes.** (XLSX 57 kb) Additional file 7:
**The up-methylated genes and functions on E60 versus E50.** (XLSX 376 kb) Additional file 8:
**The down-methylated genes and functions on E60 versus E50.** (XLSX 142 kb) Additional file 9:
**Significant pathways related with up-methylated genes and the genes involved.** (XLSX 81 kb) Additional file 10:
**Significant pathways related with down-methylated genes and the genes involved.** (XLSX 36 kb) Additional file 11:
**The genes identified by signal-net analysis.** (XLS 51 kb)

## Abbreviations

BSP: Bisulfite sequencing PCR
CDS: Coding sequence
DMR: Differentially methylated region
GO: Gene ontology
KEGG: Kyoto encyclopedia of genes and genomes
MeDIP: Methylated DNA immunoprecipitation
TSS: Transcription start site.
