# Supplementary material for: Genome-wide DNA methylation profile of developing deciduous tooth germ in miniature pigs
Source: BMC Genomics. 2016 Feb 24;17:134. doi: 10.1186/s12864-016-2485-9 (PMC4766650; doi:10.1186/s12864-016-2485-9)
Supplement: Additional file 1: Table S1. — Date generated by MeDIP-seq. Table S2 Numbers of differentially methylated regions in different gene components. Table S3 The information of primers for bisulfite sequencing. (DOCX 21 kb) [file 12864_2016_2485_MOESM1_ESM.docx]

**Table S1 Date generated by MeDIP-seq**

| sample | | Total number of reads | Total mapped reads | Percentage of mapped reads in total reads | Total unique mapped reads | Percentage of unique mapped reads（%） |
| --- | --- | --- | --- | --- | --- | --- |
| E50 | 1 | 93,877,552 | 80,303,229 | 85.55 | 59,001,337 | 62.85 |
|  | 2 | 93,877,552 | 79,227,952 | 84.41 | 56,284,467 | 59.95 |
|  | 3 | 93,877,552 | 79,124,544 | 84.30 | 57,082,119 | 60.80 |
| E60 | 1 | 93,877,552 | 79,337,684 | 84.54 | 56,560,041 | 60.24 |
|  | 2 | 93,877,552 | 79,394,355 | 84.57 | 56,715,814 | 60.42 |
|  | 3 | 93,877,552 | 79,437,772 | 84.67 | 56,790,741 | 60.49 |

**Table S2 Information of peaks**

| sample | | Total number of peaks | Peak mean length | Peak total length | Peak coverd size in genome（%） |
| --- | --- | --- | --- | --- | --- |
| E50 | 1 | 179,112 | 1151.56 | 206,257,742 | 7.94 |
|  | 2 | 144,557 | 1285.99 | 185,898,569 | 7.16 |
|  | 3 | 165,454 | 1211.13 | 200,385,895 | 7.72 |
| E60 | 1 | 135,043 | 1339.41 | 180,878,016 | 6.97 |
|  | 2 | 146,183 | 1252.63 | 183,113,912 | 7.05 |
|  | 3 | 150,082 | 1341.97 | 201,405,063 | 7.76 |

**Table S3 Numbers of differentially methylated regions in different gene components**

| gene region | Peak number | |
| --- | --- | --- |
|  | up | down |
| upstream 2K | 321 | 156 |
| 5'UTR | 161 | 33 |
| CDS | 1141 | 256 |
| Intron | 5991 | 1852 |
| 3'UTR | 101 | 45 |
| downstream 2K | 315 | 116 |

**Table S4 The information of primers for bisulfite sequencing**

| Gene region | Primer sequence | Product size (bp) |
| --- | --- | --- |
| 1 | F: GGTGGAGATAGGAAGAAGTTATT | 257 |
|  | R: AAACCCTAATTTATAACATTTACC |  |
| 2 | F: TTTGTTGTTGTTGTTGTTGTTAT | 253 |
|  | R: CCCRTCRTAATACAATAATTAAC |  |
| 3 | F: ATTATTGTTAAGTTTTATTTGTTATTT | 394 |
|  | R: AAACCCACAATACAAAATTTAAC |  |
