# Supplementary figures and images for: Genome-wide DNA methylation profile of developing deciduous tooth germ in miniature pigs
Source: BMC Genomics. 2016 Feb 24;17:134. doi: 10.1186/s12864-016-2485-9 (PMC4766650; doi:10.1186/s12864-016-2485-9)

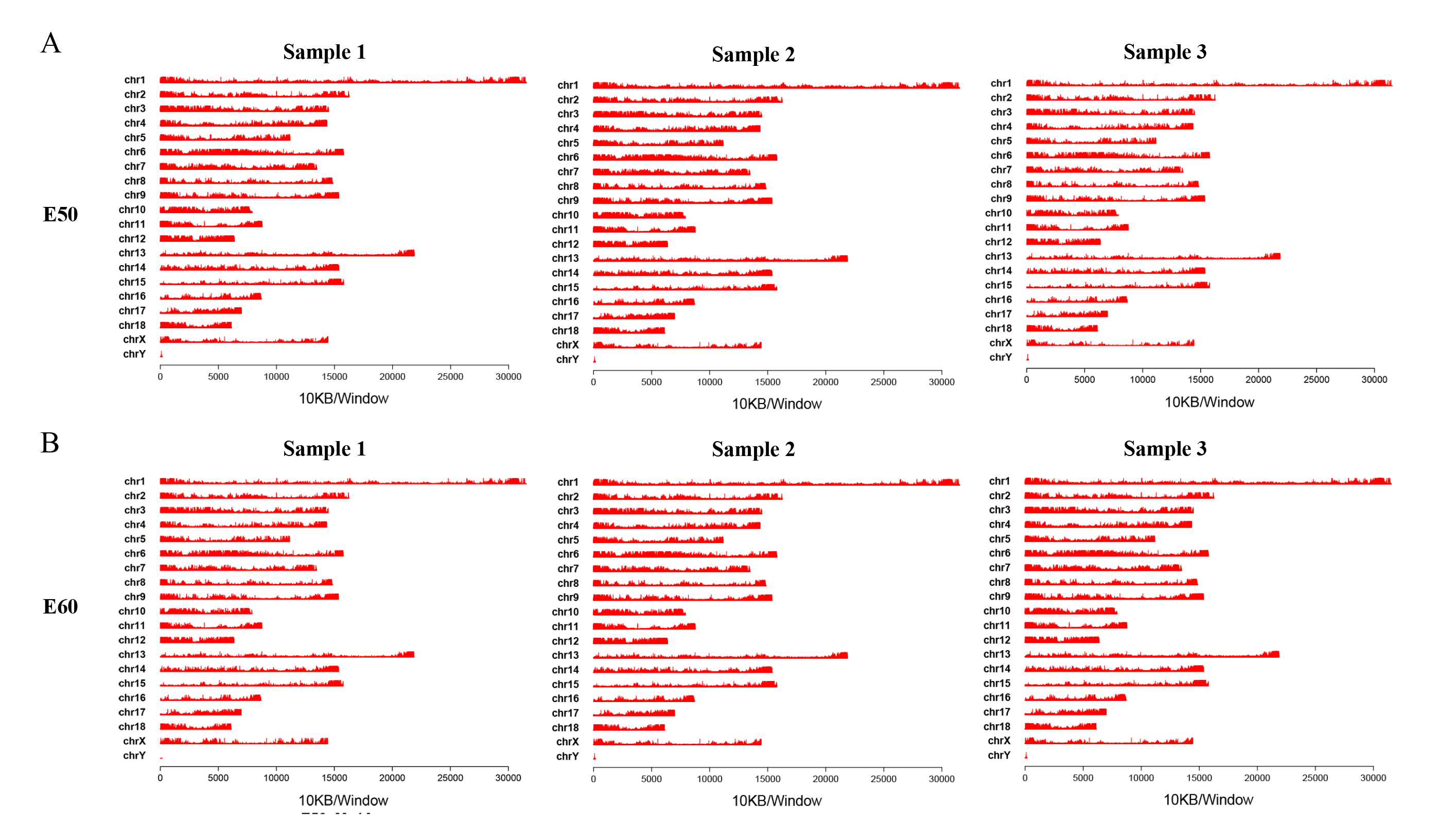

Supplement: Additional file 2: Figure S1. — Chromosome distribution of reads in the E50 and E60 tooth germ. (TIF 1845 kb) [file 12864_2016_2485_MOESM2_ESM.tif]

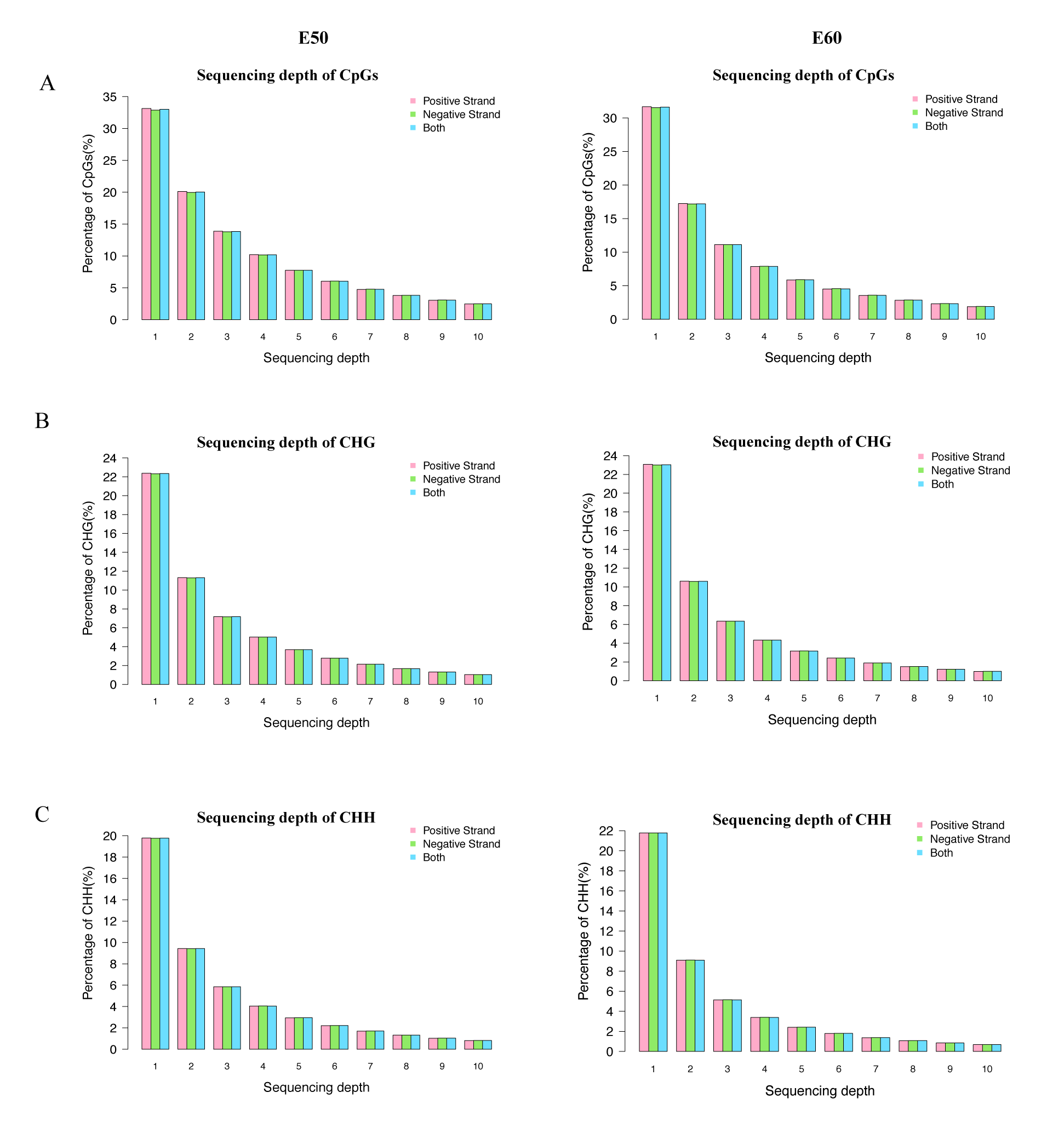

Supplement: Additional file 3: Figure S2. — Genome coverage of the CG, CHG, and CHH sites under different sequencing depth. (TIF 617 kb) [file 12864_2016_2485_MOESM3_ESM.tif]

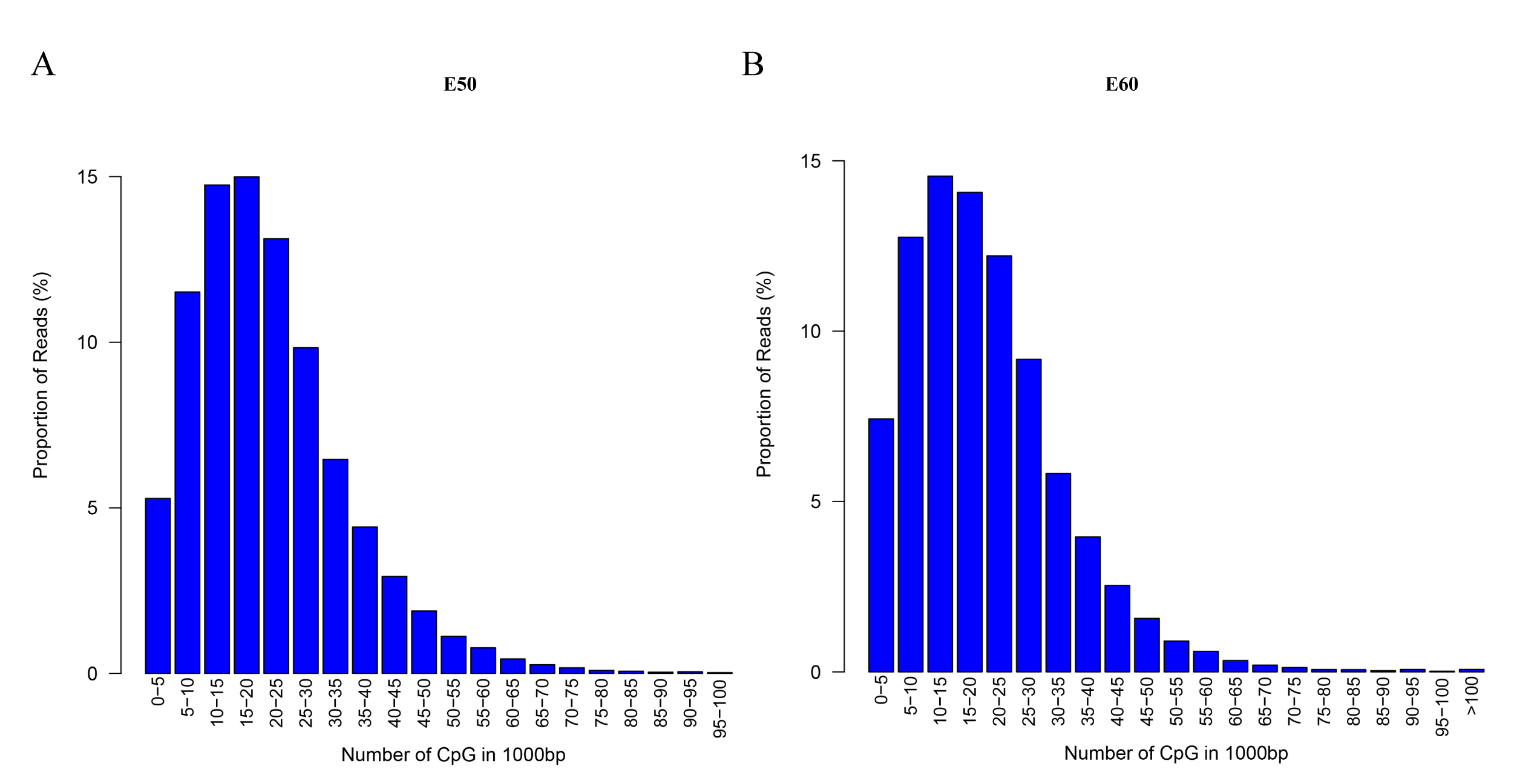

Supplement: Additional file 4: Figure S3. — Distribution of MeDIP-Seq reads in different CG density regions. (TIF 504 kb) [file 12864_2016_2485_MOESM4_ESM.tif]

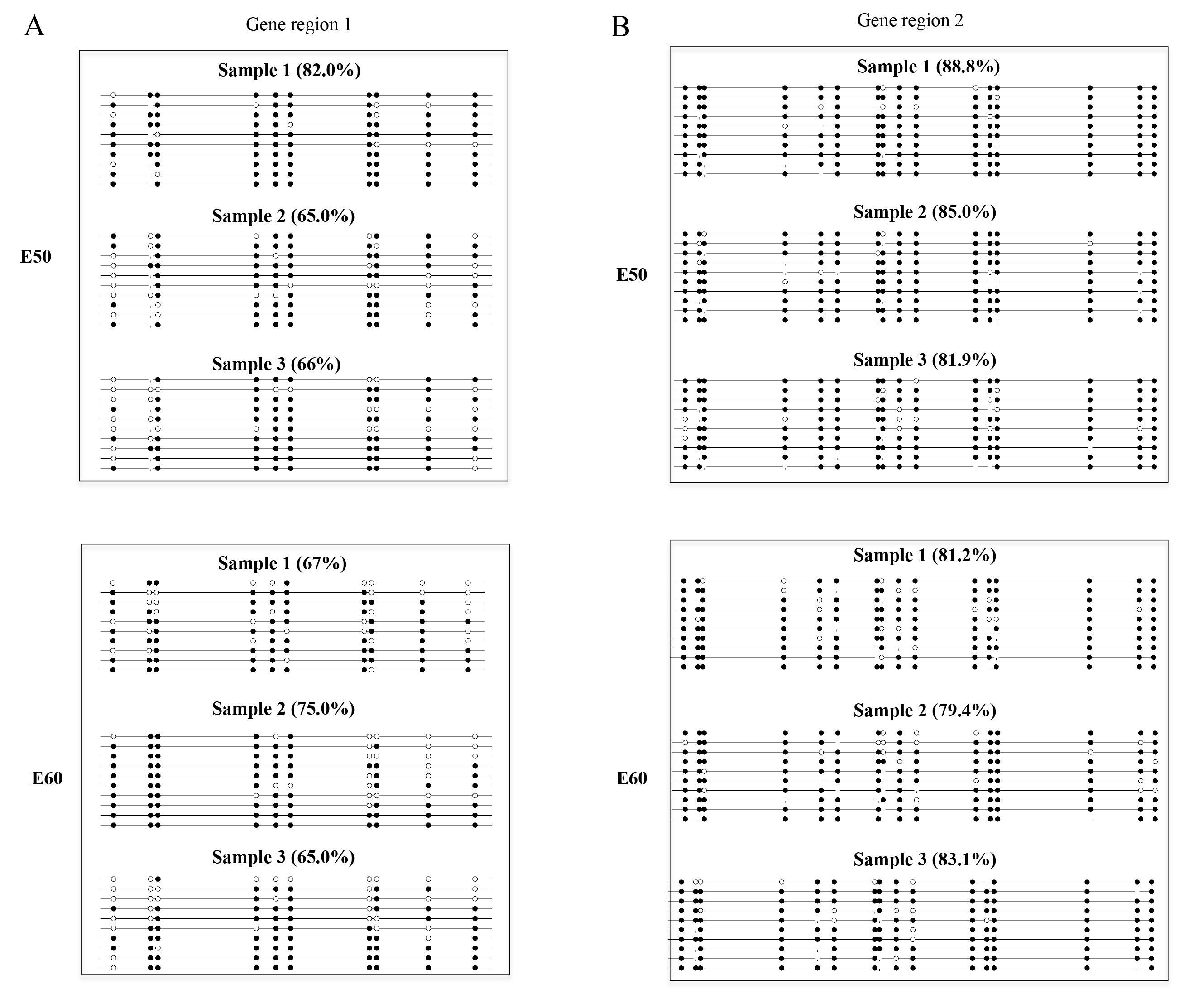

Supplement: Additional file 5: Figure S4. — The validation of MeDIP-seq data by bisulfite sequencing (BSP). (TIF 1800 kb) [file 12864_2016_2485_MOESM5_ESM.tif]
